# *TERTp* mutations and p53 expression in head and neck cutaneous basal cell carcinomas with different aggressive features

**DOI:** 10.1038/s41598-021-89906-w

**Published:** 2021-05-17

**Authors:** António Castanheira, Maria João Vieira, Mafalda Pinto, Carolina Dias, Luísa Prada, Sofia Macedo, Margarida Sá Fernandes, Fortunato Vieira, Paula Soares, Alberto Mota, José Manuel Lopes, Paula Boaventura

**Affiliations:** 1 Department of Otorhinolaryngology, Centro Hospitalar de Trás-Os-Montes e Alto Douro, Vila Real, Portugal; 2 FMUP-Faculty of Medicine, University of Porto, Porto, Portugal; 3 IPATIMUP-Institute of Molecular Pathology and Immunology, University of Porto, Porto, Portugal; 4 i3S - Instituto de Investigação e Inovação em Saúde, Universidade do Porto, Rua Alfredo Allen 208, 4200-135 Porto, Portugal; 5 Laboratory of Clinical Pharmacology and Therapeutics, Faculty of Medicine, University of Lisbon, Lisboa, Portugal; 6 Department of Pathology, Centro Hospitalar São João, Porto, Portugal; 7 HISTOCIT – Laboratório de Anatomia Patológica, Trofa, Portugal; 8 Department of Dermatology, Centro Hospitalar São João, Porto, Portugal

**Keywords:** Cancer, Cancer genetics, Skin cancer

## Abstract

Cutaneous basal cell carcinoma (cBCC) is an economic burden to health services, due to its great morbidity and increasing incidence in old people. Infiltrative cBCCs and cBCCs with micronodular pattern are considered as more aggressive. The role of p53 expression and *TERTp* mutation on cBCC behavior remains to be clarified. We aimed to assess *TERTp* mutations and p53 expression in relation to the cBCC histological subtype in a cohort of patients referred to an ENT Department of a tertiary Hospital of Northern Portugal. We performed a retrospective clinicopathological and histological review of the head and neck cBCCs followed-up at the otorhinolaryngology department of Trás-os-Montes e Alto Douro hospital (January 2007–June 2018). We assessed *TERTp* mutations in 142 cBCCs and p53 protein expression, through immunohistochemistry, in 157 cBCCs. We detected *TERTp* mutations in 43.7% of cBCCs and p53 overexpression in 60.5% of cBCCs. We spotted association of p53 overexpression and *TERTp* mutation with necrosis. In the infitrative-growth pattern cBCCs, there was no significant association with the clinical and histological features evaluated, except for necrosis. In the indolent-growth cBCCs, we identified a significant association of *TERTp* mutation status with female sex, necrosis, multiple cBCCs, and p53 positive expression. Our results suggest that *TERTp* mutation may be useful to identify more aggressive features in the indolent-growth pattern cBCCs (nodular and superficial subtypes). Further studies with larger cohorts are warranted to clarify the relevance of *TERTp* mutation in cBCCs.

## Introduction

Cutaneous basal cell carcinoma (cBCC) is the most common cancer diagnosed in human populations, representing 70–80% of skin cancers^1,2^. Despite its very low mortality rate, it is an economic burden to health services, due to its great morbidity and increasing incidence in old people^1,3^. The main carcinogenic agent is ultraviolet light (UV), which is reflected by the cBCCs higher frequency in sun-exposed sites^2^.

cBCC is characterized by great morphological variability, both clinically and histologically^4^. cBCCs can be classified in three main histopathological subtypes: nodular, superficial, and infiltrative^5^, but cBCC with mixed histology is a common pattern^1^. cBCCs can present indolent or aggressive behavior^6^. Superficial and nodular cBCCs subtypes have a more indolent behavior whereas infiltrative subtypes and BCCs with micronodular features are usually more aggressive^6,7^. cBCCs exhibiting infiltrative growth display a higher propensity for recurrence than well-circumscribed cBCCs so the histological subtyping is crucial for evaluating the risk of relapse^4^.

Several genes were associated with cBCC, including the key components of the Hedgehog pathway, *PTCH*1 and *SMO*, the *TP*53 tumor suppressor, and members of the *RAS* proto-oncogene family^3^. The *PTCH* gene is the most commonly mutated, involved in 90% of cBCCs^4^. Inactivation of the *TP*53 gene is also a frequent event associated with cBCC pathogenesis^3^. p53 expression and its expression in actinic keratosis suggests that p53 plays a role in the early steps of carcinogenesis in skin cancers^8^. Moreover, it has been suggested that p53 expression seems to be associated with cBCC aggressiveness^9–12^, and some differences in p53 mutation frequency, types of mutation, and hot spots were seen between aggressive and nonaggressive BCCs^13^. However, these differences do not constitute clear-cut diagnostic or prognostic indicators of tumor aggressiveness, so tumor aggressiveness may be attributable to other genetic events that occur during tumor progression^13^.

Recent reports described non-coding mutations in the *TERT* promoter gene (*TERT*p) as a frequent event in cBCCs^14–16^. Mutations in the promoter region of *TERT* lead to increased *TERT* expression and de novo telomerase activity in cancer cells, which allow cells to acquire the ability to overcome senescence and to become immortal^17^. Telomerase up-regulation is a key, rate-limiting step in tumorigenesis as evidenced by the highly recurrent mutations in the TERT promoter^18^. Inactivating mutations in the p53 pathway and activating mutations in the TERT promoter may be critical in enabling tumor progression^18^. *TERTp* mutations have been associated with poor prognosis in several cancers including melanoma and squamous cell carcinoma^15,19,20^ but, so far, this has not been observed in BCC. Additionally, there are no reports describing an association of the genetic profile with specific histopathological cBCCs subtypes, namely the more aggressive infiltrative subtype.

Taking this into account, we aimed to assess p53 expression and *TERTp* mutation status in cBCC, and associate them with histological subtype and clinicopathological features, in a cohort of cBCC patients.

## Material and methods

### Cases studied

Selection criteria for inclusion in this retrospective series were all cases of histologically confirmed cutaneous BCCs (cBCCs) located in head and neck, managed at the ENT Department of the Centro Hospitalar de Trás-os-Montes e Alto Douro (CHTMAD), from January 2007 to June 2018. Exclusion criteria included age < 18 year-old, genetic diseases or syndromes predisposing to cBCC, genetic disorders associated with other neoplasms, diffuse dermatosis, and biopsies from recurrence or persistence of a previously diagnosed cBCC. Applying these criteria, 151 patients were included and signed an informed consent after proper information about the goal and scope of the study. Data from these patients were gathered in a personal interview questionnaire, using medical records (*SClinico* database and Clinical Process), and pathological records from the Pathology Department at CHTMAD. The Ethical Committee and the Administrative Council of CHTMAD approved the study in 16/10/2012. All the methods were performed in accordance with relevant guidelines and regulations.

Patient melanin content in the forehead and inner arm was measured with a DermaSpectrometer (Cortex Technology, Hadsund, Denmark) as described by Shriver and Parra^21^. Sun-exposure was assessed according to the patient’s profession and lifestyle—high exposure was considered for patients who had professions with many hours of sun exposure such as farmers and construction workers.

Patients with previous incomplete or inappropriate histological records, missing information, or histological samples in inappropriate conditions were excluded (n = 55), so we ended up with 96 patients. Of these 96 patients, 36 had more than one BCC. The final series comprised 160 cBCCs from 96 patients.

cBCC histopathological features were reviewed by a pathologist (MSF), under the supervision of a senior pathologist/dermatopathologist (JML), to assess detailed histological data for each tumor. The morphological classification of cBCCs was performed according to Rippey^22^ and included the following growth patterns: nodular, infiltrative (including morpheic), superficial or mixed (including a combination of at least two of the aforementioned patterns). The cBCCs were further classified as cBCCs with an infiltrative-type growth pattern, considered as more aggressive, which included BCCs with infiltrative, morphoeform or micronodular component, and BCCs with an indolent-type growth pattern, considered as less aggressive, which included the nodular and superficial subtypes^6,7^.

### p53 immunohistochemistry

p53 protein expression in cBCCs was evaluated through immunohistochemistry. Briefly, deparaffinized and rehydrated sections were subjected to a 45’ steamer treatment in 10 mM sodium citrate buffer, pH 6.0, for antigen retrieval. Ultravision hydrogen peroxide block (Ref TA-060-H202Q, Thermo Scientific) and protein block (Ref TA-125-PBQ, Thermo Scientific) were used to block endogenous peroxidase, 10’ each. Subsequently, we used the monoclonal antibody anti-p53 (NCL-L-p53, Leica), diluted 1:550 (Ref TA-125-ADQ, Thermo Scientific), for 60’ at room temperature. The monoclonal antibody (clone DO-7) recognizes both wild type and mutant forms of human p53 protein under denaturing and non-denaturing conditions. After this step, sections were processed for the detection of positive immunohistochemical reaction with HRP polymer quantum (Ref TL-060-QPH, Thermo Scientific) for 10’, and further reaction with 3% diaminobenzidine chromogen (DAB, Ref K3468, Dako), for 3’. Finally, sections were counterstained with Mayer’s hematoxylin, cleared and mounted.

For the p53 staining evaluation, the slides were digitalized using the scanner D-sight FLUO (A-MENARINI diagnostics, UK) and its automatized analysis program with a previously developed p53 algorithm. Approximately 2000 cells were counted in each cBCC.

The scoring of tumors was done as follows: staining < 10% of tumor cells were considered as negative, and staining ≥ 10% was considered as positive^23,24^.

### Molecular analysis: TERTp mutations

*TERTp* mutation analysis was performed as previously described^15,25^. Briefly, cBCCs areas were manually dissected from 10-µm whole sections of paraffin-embedded material. DNA extraction was performed using with GRiSP kit (GRiSP, Portugal) according to the manufacturer instructions. After the last centrifugation, DNA was quantified with the Nanodrop (ND-1000, Thermo Fisher Scientific, Lithuania). Mutation analysis was performed with Sanger sequencing. Briefly, genomic DNA (25–100 ng) was amplified by polymerase chain reaction (PCR) with the kit from Bioline (MyTaq HS Mix 2X, USA), using the following set of primers: forward *TERTF*, CAGCGCTGCCTGAAACTC; and reverse *TERTR*, GTCCTGCCCCTTCACCTT. The following cycling conditions were used: 35 s at 94 °C; 40 s at 62 °C and 45 s at 72 °C for 40 cycles. Products were enzymatically purified with Exonuclease I and Shrimp Alkaline Phosphatase and sequenced in an ABI Prism 3130 xl Automatic sequencer (Perkin-Elmer, Foster City, CA) using the BigDye v3.1 Sequencing Kit (Applied Biosystems, Washington). Cases with mutations were confirmed by an independent PCR amplification. Additionally, randomly selected cases were confirmed using real-time PCR amplification curve analysis^25^. Positive and negative mutation control samples were included in each run to ensure the assay’s validity.

### Statistical analysis

Statistical analysis was performed using the statistical package for social sciences software (IBM SPSS Statistics 23). Proportions were compared using the X^2^ test or Fisher’s exact test when appropriate (Yates correction in case of multiple entries); the significance of differences between means was assessed with Student’s unpaired t-test. A *p *value < 0.05 was considered statistically significant with a 95% confidence interval.

### Ethics approval

The Ethical Committee and the Administrative Council of CHTMAD approved the study in 16/10/2012.

### Consent to participate

Patients signed an informed consent after proper information about the goal and scope of the study.

## Results

### p53 immunostaining

**Figure 1:**
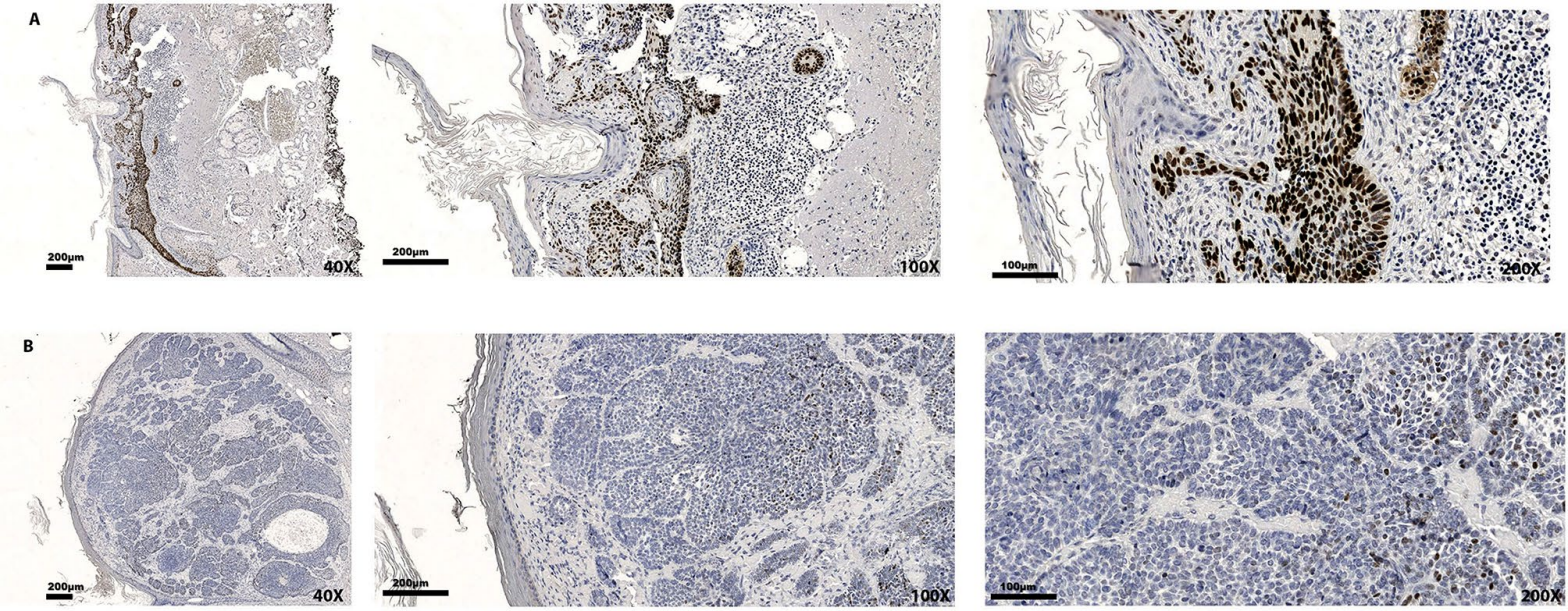
Representative examples of p53 immunoexpression in cBCCs: (**A**) positive (90.6%) expression; (**B**) negative (2.3%) expression.

p53 positive staining (≥ 10% of the stained tumor cells) was observed in the nucleus of 95 out of 157 cBCCs (60.5%; in three cases the evaluation was not possible due to unspecific staining) (Fig. 1). None of the cases displayed either absence of expression or cytoplasm expression of p53 in the tumor cells. No significant differences were observed concerning age at diagnosis, sex, skin melanin content in inner arm, solar exposure, and presence of multiple cBCCs, between p53 positive and p53 negative cBCCs (Table 1).

**Table 1:** Clinicopathological features and p53 expression in cBCCs.

Clinicopathological features	Total cBCC	p53 negative (< 10%)	p53 positive (≥ 10%)	*p* value
**Age at diagnosis (mean ± SD) (n = 157)**	73.0 ± 11.2	72.9 ± 12.0	73.1 ± 10.7	0.905
**Sex [n (%)](n = 157)**				
Male	82 (52.2)	35 (56.5)	47 (49.5)	0.467
Female	75 (48.8)	27 (43.5)	48 (50.5)	
**Skin melanin content (inner arm) (mean ± SD) (n = 124)**	28.3 ± 3.3	28.2 ± 2.8	28.3 ± 3.5	0.773
**Sun exposure (mean ± SD) (n = 134)**				
Low	34 (25.4)	13 (25.5)	21 (25.3)	0.981
High	100 (74.6)	38 (74.5)	62 (74.7)	
**Location [n (%)] (n = 157)**				
Nose	39 (24.9)	14 (22.6)	25 (26.3)	0.997
Face	47 (29.9)	19 (30.6)	28 (29.5)	
Eyelid	31 (19.8)	14 (22.6)	17 (17.9)	
Preauricular / auricular region	27 (17.2)	9 (14.5)	18 (18.9)	
Scalp	1 (0.6)	1 (1.6)	0 (0)	
Lip	6 (3.8)	3 (4.8)	3 (3.2)	
**Tumor dimension (mean ± SD) (n = 154)**	13.2 ± 9.8	13.3 ± 8.1	13.2 ± 10.8	0.987
**Focality [n (%)] (n = 157)**				
Single	61 (38.9)	24 (38.7)	37 (38.9)	0.976
Multiple	96 (61.1)	38 (61.3)	58 (61.1)	

Concerning cBCCs histopathological features (tumor thickness, tumor dimension, location, histological subtype, invasion level, ulceration, associated actinic keratosis, necrosis, tumor pigmentation, elastosis, and growth pattern), no significant differences were found according to p53 positive expression except for necrosis, which was a more frequent finding in the p53 positive cBCCs (25.5%) comparing with the negative ones (11.7%) (*p* = 0.036) (Table 2).

**Table 2:** cBCCs histopathological features and p53 expression.

Histopathological features	Total cBCC	p53 negative (< 10%)	p53 positive (≥ 10%)	*p* value
**Tumor thickness (mean ± SD) (n = 151)**	2.4 ± 1.7	2.4 ± 1.8	2.3 ± 1.5	0.713
**Histological subtype [n (%)] (n = 157)**				
Nodular	56 (35.7)	24 (38.7)	32 (33.7)	0.444
Infiltrative	51 (32.5)	20 (32.3)	31 (32.6)	
Mixed	44 (28.0)	16 (25.8)	28 (29.5)	
Superficial	6 (3.8)	2 (3.2)	4 (4.2)	
**Invasion level [n (%)] (n = 152)**				
Papillary dermis	8 (5.2)	2 (1.3)	6 (6.5)	0.547
Reticular dermis	84 (55.3)	30 (50.0)	54 (58.7)	
Subcutaneous	58 (38.2)	26 (43.3)	32 (34.8)	
Intramuscular	2 (1.3)	2 (3.3)	0 (0)	
**Tumor features [n (%)]**				
Ulceration (n = 153)	95 (62.1)	34 (58.6)	61 (64.2)	0.489
Actinic keratosis (n = 152)	14 (9.2)	4 (6.8)	8 (8.6)	0.767
Necrosis (n = 154)	31 (20.1)	7 (11.7)	24 (25.5)	**0.036**
Pigmentation (n = 154)	14 (9.1)	8 (13.3)	6 (6.4)	0.143
Elastosis (n = 153)	14 (9.2)	6 (10.0)	8 (8.6)	0.77
**Lymphocytic infiltrate [n (%)] (n = 156)**				
Absent/Rare	82 (52.6)	34 (55.7)	48 (50.5)	0.525
Moderate/Intense	74 (47.4)	27 (44.3)	47 (49.5)	
**Lymphovascular invasion [n (%)](n = 150)**				
Not identified	145 (96.7)	56 (94.9)	89 (97.8)	0.807
Present	5 (3.3)	3 (5.1)	2 (2.2)	
**Perineural invasion [n (%)] (n = 152)**				
Not identified	142 (94.0)	56 (93.3)	86 (94.5)	0.967
Present	9 (6.0)	4 (6.7)	5 (5.5)	
**Growth pattern [n (%)] (n = 157)**				
Indolent-type	56 (35.7)	22 (35.5)	34 (35.8)	0.969
Aggressive-type	101 (64.3)	40 (64.5)	61 (64.2)	

Bold - significant difference (*p*-value < 0.05).

Additionally, we repeated the same analysis separately for cBCC with infiltrative-type growth pattern *vs* indolent-type growth pattern, as this classification reflects BCC aggressiveness. In cBCCs with indolent-type growth pattern, we observed a significant association of p53 expression with *TERTp* mutation, which will be described in the next section. We also observed an association of necrosis with p53 expression in this subgroup, which had been already observed when analyzing all the cBCCs together. A higher frequency of necrosis was observed in cBCCs with positive p53 expression (11/34, 32.4%) comparing with p53 negative cBCCs (1/22, 4.5%) (p = 0.018). No significant associations were found in the infiltrative-type growth pattern group.

### TERTp mutations

**Figure 2:**
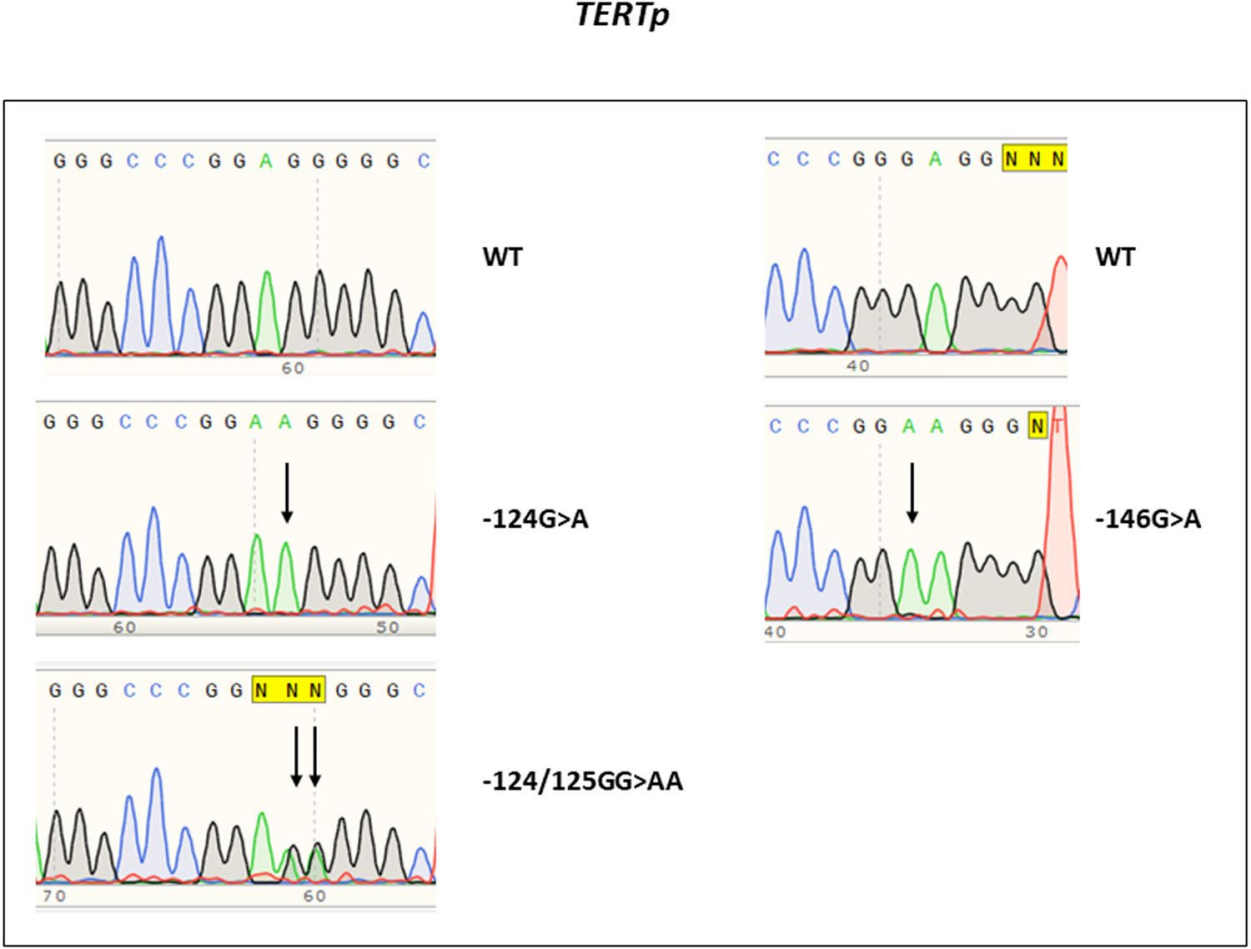
Electrophoregram with representative examples of the *TERTp* mutations observed.

We attained information on *TERTp* mutation status in 142 cBCCs of the 160 cBCCs evaluated (88.8%); in 18 cases, we could not obtain enough DNA or DNA quality to ensure a proper amplification. In these 142 cases we did not achieve p53 evaluation in threes cases, meaning we had 139 cases with evaluation of both, *TERTp* mutations and p53 expression. In the142 cBCCs, 62 were *TERTp* mutated (43.7%). The most frequent mutation was the − 146:G > A mutation, which occurred in 48 of 62 cases (77.4%). The− 124:G > A occurred in 13 out of 62 cases (21.0%) (Fig. 2). In one cBCC, we observed the tandem mutation − 124/125:GG > AA (Fig. 2), and in another cBCC the − 124:G > A and − 146:G > A simultaneous mutations were observed (1.6%).

No significant differences were observed concerning age at diagnosis, sex, skin melanin content in the forearm, solar exposure, and presence of multiple cBCCs, between *TERTp* mutated and *TERTp* wild type (wt) cBCCs (Table 3).

**Table 3:** Clinicopathological features in cBCCs and *TERTp* mutation status.

Clinicopathological features	Total cBCCs (n = 142)	*TERT*wt (n = 80)	*TERT*mut (n = 62)	*p* value
Age at diagnosis (mean ± SD) (n = 142)	72.4 ± 11.2	72.4 ± 11.1	72.7 ± 11.3	0.887
**Sex [n (%)] (n = 142)**				
Male	78 (54.9)	46 (57.5)	32 (51.6)	0.484
Female	64 (45.1)	34 (42.5)	30 (48.4)	
**Skin melanin content (inner arm) (mean ± SD) (n = 114)**	28.3 ± 3.3	28.3 ± 3.7	28.2 ± 2.8	0.924
**Sun exposure (mean ± SD) (n = 121)**				
Low	30 (24.8)	17 (25.4)	13 (24.1)	0.869
High	91 (75.2)	50 (74.6)	41 (75.9)	
**Location [n (%)] (n = 142)**				
Nose	31 (21.8)	17 (21.3)	14 (22.6)	0.876
Face	43 (30.3)	23 (28.7)	20 (32.3)	
Eyelid	30 (21.1)	21 (26.3)	9 (14.5)	
Preauricular/Auricular region	26 (18.3)	14 (17.5)	12 (19.4)	
Scalp	1 (0.7)	0 (0)	1 (1.6)	
Lip	5 (3.5)	3 (3.8)	2 (3.2)	
Other	6 (4.2)	2 (2.5)	4 (6.5)	
**Tumor dimension (mean ± SD) (n = 142)**	13.6 ± 10.1	14.0 ± 11.7	13.1 ± 7.7	0.612
Other				
**Focality [n (%)] (n = 142)**				
Single	56 (39.4)	35 (43.8)	21 (33.9)	0.232
Multiple	86 (60.6)	45 (56.2)	41 (66.1)	

Concerning cBCCs histopathological features (tumor thickness, tumor dimension, location, histological subtype, invasion level, ulceration, associated actinic keratosis, skin pigmentation, elastosis, and growth pattern) no significant differences were found according to the *TERTp* mutation status (Table 4), except for a significant association with necrosis, which was more frequent in *TERTp* mutated cBCCs (33.9%), compared with the *TERTp* wt cBCCs (10.3%) (*p* = 0.001). No association was found between p53 expression and *TERTp* mutation status in this cohort of cBCCs.

**Table 4:** cBCCs histopathological features and *TERTp* mutation status.

Histopathological features	Total cBCCs (n = 142)	*TERT*wt (n = 80)	*TERT*mut (n = 62)	*p* value
Tumor thickness (mean ± SD) (n = 136)	2.44 ± 1.72	2.21 ± 1.57	2.73 ± 1.87	0.081
**Histological subtype (n = 142)**				
Nodular	52 (36.6)	25 (31.3)	27 (43.5)	0.095
Infiltrative	45 (31.7)	30 (37.5)	15 (24.2)	
Mixed	39 (27.5)	19 (23.8)	20 (32.3)	
Superficial	6 (4.2)	6 (7.5)	0 (0)	
**Invasion level**				
Papillary dermis	7 (5.1)	7 (9.2)	0 (0)	0.178
Reticular dermis	76 (55.1)	42 (55.3)	34 (54.8)	
Subcutaneous	53 (38.4)	26 (34.2)	27 (43.5)	
Intramuscular	2 (1.4)	1 (1.3)	1 (1.6)	
**Tumor features**				
Ulceration (n = 139)	50 (36.0)	27 (34.6)	23 (37.7)	0.706
Actinic keratosis (n = 139)	11 (7.9)	7 (9.0)	4 (6.6)	0.755
Necrosis (n = 140)	29 (20.7)	8 (10.3)	21 (33.9)	**0.001**
Pigmentation (n = 140)	13 (9.3)	9 (11.5)	4 (6.5)	0.303
Elastosis (n = 139)	13 (9.4)	9 (11.7)	4 (6.5)	0.292
**Lymphocytic infiltrate (n = 142)**				
Absent/Rare	76 (53.5)	39 (48.8)	37 (59.7)	0.195
Moderate/Intense	66 (46.5)	41 (51.2)	25 (40.3)	
**Lymphovascular invasion (n = 136)**				
Not identified	132 (97.1)	71 (94.7)	61 (100)	0.187
Present	4 (2.9)	4 (5.3)	0 (0)	
**Perineural invasion (n = 137)**				
Not identified	128 (93.4)	70 (92.1)	58 (95.1)	0.725
Present	9 (6.6)	6 (7.9)	3 (4.9)	
**Growth pattern (n = 142)**				
Indolent-type	51 (35.9)	32 (40.0)	19 (30.6)	0.249
Infiltrative-type	91 (64.1)	48 (60.0)	43 (69.4)	

Bold - significant difference (*p*-value < 0.05).

The significant association with necrosis, observed when we considered the whole cBCCs analyzed, persisted when the groups were split according to the growth pattern. In cBCCs with infiltrative-type growth pattern, necrosis was present in 12 out of 43 *TERTp* mutated cBCCs (27.9%), compared with 5 out of 46 *TERTp* wt cBCCs (10.9%) (*p* = 0.041). No other significant associations were found in the infiltrative-type growth pattern group. In cBCCs with indolent-type growth pattern, significant associations were observed for *TERTp* mutation status and female sex, multiple cBCC, and p53 positive expression (Table 5).

**Table 5:** Features significantly associated with *TERTp* mutation status in indolent-type growth pattern cBCCs.

Features	Total (n = 51)	wt (n = 32)	*TERTp* (n = 19)	*p* value
**Sex [n (%)]**				
Male	23 (45.1)	18 (56.3)	5 (26.3)	**0.038**
Female	28 (54.9)	14 (43.7)	14 (73.7)	
**Focality [n (%)]**				
Single	20 (39.2)	16 (50.0)	4 (21.1)	**0.041**
Multiple	31 (60.8)	16 (50.0)	15 (78.9)	
**p53**				
p53 negative (< 10%)	16 (38.0)	3 (15.8)	13 (46.4)	**0.020**
p53 positive (≥ 10%)	31 (62.0)	16 (84.2)	15 (53.6)	
**Necrosis**				
Absent	38 (74.5)	29 (90.6)	10 (52.6)	**0.005**
Present	23 (23.5)	3 (9.4)	9 (47.4)	

Bold - significant difference (*p*-value < 0.05).

Finally, we compared p53 positive and *TERTp* mutated cases, with p53 negative and *TERTp* wild type cases (data not shown). The only significant difference found was the frequency of necrosis. cBCCs p53 positive and *TERTp* mutated had more frequently necrosis (17/ 37, 45.9%) when compared with cBCCs p53 negative and *TERTp* wild type (4/31, 12.9%) (*p* = 0.003).

## Discussion

In the present study, *TERTp* mutations and p53 expression were evaluated in cBCCs from patients referred to an ENT Hospital Department. We observed that 32% of the cBCCs were of the infiltrative subtype, which is considered as more aggressive due to its higher risk of subclinical tumor extension^26^. In most series, lower frequencies of infiltrative cBCC were reported, ranging from 4.2 to 8.7%^27–32^. Leibovitch et al*.* reported a higher frequency, 28.3%, which they ascribed to a referral bias for Micrographic Mohs Surgery (MMS)^33^. The second most frequent subtype detected in the present study was the mixed cBCC subtype. Again, this may be related to a more aggressive pattern, since mixed type cBCCs have been reported as more frequently composed of aggressive subtypes^34^.

The cBCC location distribution of our series, with the face and nose as the main locations, is in line with what was been reported in other studies^30,33,35^. A relevant proportion of the cBCCs invaded the deep skin layers (reticular dermis and subcutaneous layer) when compared with other studies^35–37^. Indeed, Bandeira et al*.* did not report invasion deeper than the reticular dermis^36^, but Betti et al*.* reported subcutaneous fat invasion in infiltrative cBCCs, including the micronodular subtype^37^. Our results can be related to the presence of a high proportion of the infiltrative subtype since infiltrative cBCCs usually invade deeper than nodular cBCCs^36–38^.

Multiple cBCCs were more frequent in our series (38.9%) than the 16% reported by Scrivener et al*.*^29^ in the largest BCCs series described to date. In previous studies from our group, we found a frequency ranging from 30.0 to 38.1%^15,32^. Multiple cBCCs are a sign of aggressiveness as they increase the risk of relapse^2^.

In our study, most cBCCs overexpressed the p53 protein (60.5%), a frequency higher than the 45% found by Stamatelli et al.^23^ but similar to the values observed by Haghighi et al.^24^; both authors used the ≥ 10% cut-off to consider p53 positive expression. No associations were found between p53 overexpression and the clinical and histological features of the cBCCs, except for necrosis. Necrosis was significantly more frequent in cBCCs with p53 overexpression. Information about necrosis as a prognosis factor in BCC is scarce. Welsch et al*.*^38^ observed this feature in 61% of their cBCC series and reported that cBCCs without necrosis had a significantly deeper invasion than those with necrosis. Recent studies revealed a role for p53 in regulating necrotic cell death by activating independent signaling pathways that include induction of mitochondrial outer and inner membrane permeability, and altered mitochondrial dynamics^39^.

It has been reported that p53 overexpression is significantly higher in aggressive cBCCs compared with the non-aggressive ones^10–12^. Our data do not corroborate this finding since cBCCs with indolent-type or infiltrative–type growth pattern displayed an equivalent frequency of p53 overexpression. Furthermore, other authors reported that overexpression of p53 does not always reflect the degree of malignancy in cutaneous neoplasms^40^.

Regarding *TERTp* mutations, 42% of the cBCCs were mutated, a lower frequency than the previously reported (51–78%)^14–16^. The most frequent mutation was the − 146:G > A, which occurred in 79% of the cases. In a previous study from our group, a similar frequency of the − 146:G > A mutation was detected in a cohort of cBCCs occurring after irradiation in childhood for *tinea capitis* (75%), but in the sporadic context (non-irradiated control group) only 25% of the mutated cBCCs harbored this mutation^15^. Other studies, such as the one from Griewank et al*.*^16^ reported a 55.6% frequency of the − 146:G > A mutation. Skin cancers seem to be the only cancers where the − 146:G > A mutation is more common than − 124:G > A mutation^20^. One cBCC harbored concurrently the − 124:G > A and the − 146:G > A mutations, and other harbored the tandem mutation at position − 124/− 125, rare events previously reported by our group^15^.

Griewank et al*.* found no statistically significant associations of *TERTp* mutation with cBCC clinical and histopathologic features^16^, nor did our group in previous studies^15^. Noteworthy, in the present study, we observed an association of *TERTp* mutation with necrosis, which also occurred for p53 overexpression.

*TERTp* mutation has not been reported as a prognostic factor in cBCCs^14–16^, at variance with other skin cancers, such as squamous cell carcinoma^41^ and melanoma^18,33^ in which *TERTp* mutations were associated with an ominous outcome. Contrarily, in the bladder cancer model, the − 146:G > A was an independent predictor of nonrecurrence after BCG therapy in the BCG-NMI tumors^42^.

We have compared p53 positive and *TERTp* mutated cases, with p53 negative and *TERTp* wild type cases (data not shown), observing that cases with concomitantly p53 positive and *TERTp* mutated did not present more aggressive features, as it could be anticipated. Again, the only significant association found was with necrosis.

Then, we decided to evaluate the cBCCs according to the type of growth pattern (indolent *vs* infiltrative), and we found that in the infiltrative-type cBCCs, considered to be the most aggressive, the only feature associated with the *TERTp* mutation was necrosis. In the less aggressive indolent-type growth pattern cBCCs, besides necrosis, there was a significant association of *TERTp* mutation with female sex, multiple cBCC, and p53 positive expression. Contrarily to what happened when considering all cBCCs together, where no association between p53 positivity and *TERTp* mutation was observed, this association was observed in the indolent-type growth pattern cBCCs.

Few studies have evaluated the concomitant presence of p53 and *TERTp* mutations, none in BCC. In solid fibrous tumours, Machado et al.^43^ reported that tumours with TP53 and TERTp mutations were almost always classified as high risk, and the patients developed metastases and/or died of the disease. Morevover, Akaike et al. reported that TP53 mutations, which result in its overexpression, in combination with TERT promoter mutations seem to play an important role in the dedifferentiation process in these tumours^44^. In thyroid cancer, the concomitant presence of TERTp and TP53 mutations may be useful for the identification of more aggressive tumours^45,46^. In the hepatocellular carcinoma model, Shulze et al. found that TERTp mutations were early event in tumour progression whereas TP53 alterations appeared at more advanced stages in aggressive tumors^47^. In urogenital cancer, Wu et al. have observed a significant co-occurrence of mutations between the TERT promoter and the tumor protein 51/retinoblastoma1 (TP53/RB1) signaling pathway, indicating that they may cooperatively contribute to the genesis and progression of bladder cancer^48^. Overall, these data show that the concomitant presence of TERTp and p53 mutations is these tumours is associated with a more aggressive pattern. Although this kind of associations were not described in cBCC, p53 positive expression, and presence of multiple cBCCs, are features independently related to cBCCs aggressiveness^10,11,22,49^.

This study has limitations which do not allow establishing *TERTp* as a new prognostic marker for cBCCs. First, we would need to have a validation cohort, which was not possible in the present study. Second, additional experiments would be needed to establish the association between p53 over expression and *TERTp* mutation.

In conclusion, it seems that *TERTp* mutation may potentially be useful to discriminate more aggressive indolent-type growth pattern cBCCs, but further studies with larger cohorts are warranted to clarify the relevance of *TERTp* mutations in cBCCs.

## Date availability

Full date will be made available upon request to the corresponding author.
